# Safety of dobutamine stress cardiovascular magnetic resonance in patients with prior coronary artery bypass grafting

**DOI:** 10.1016/j.jocmr.2024.101119

**Published:** 2024-10-28

**Authors:** Jannick Heins, Janek Salatzki, Anne Köhrer, Andreas Ochs, Lukas D. Weberling, Hauke Hund, Evangelos Giannitsis, Norbert Frey, Dirk Loßnitzer, Florian André, Henning Steen

**Affiliations:** a Department of Cardiology, Angiology and Pneumology, Heidelberg University Hospital, Heidelberg, Germany; b DZHK (German Centre for Cardiovascular Research), Partner Site Heidelberg/Mannheim, Heidelberg, Germany; c GECKO Institute, Heilbronn University of Applied Sciences, Heilbronn, Germany; d MEDNEO, Hamburg, Germany

**Keywords:** Dobutamine, CMR, Safety, Coronary artery bypass grafting

## Abstract

**Background:**

Patients with coronary artery bypass grafts (CABG) face an elevated risk of major adverse cardiac events. High-dose dobutamine stress cardiovascular magnetic resonance (DCMR) imaging is a well-established technique to detect hemodynamically significant coronary artery disease. However, there is a lack of data regarding the safety of DCMR in patients with CABG. This study aims to evaluate the safety of DCMR in patients with CABG.

**Methods:**

We retrospectively studied patients after CABG who subsequently underwent DCMR between November 2008 and July 2018. Side effects, defined as adverse events and minor symptoms, during DCMR were analyzed and compared to 200 individuals matched for age, sex, and body mass index without prior CABG undergoing DCMR.

**Results:**

Three hundred and thirty-six patients (70 ± 9 years, 85% men (284/336)) were identified. Adverse events occurred in 35 CABG patients (10% (35/336)) and 18 controls (9% (18/200), p = 0.595). A drop of systolic blood pressure (SBP) >40 mmHg (12 patients), non-sustained ventricular tachycardia (6 patients), increase in SBP >200 mmHg (5 patients), monomorphic premature ventricular contractions (PVC) (2 patients), bigeminy (2 patients), left bundle-branch block (2 patients), as well as tachycardiac paroxysmal atrial fibrillation, bradycardia, supraventricular tachycardia, couplets/triplets, and sinus arrhythmia in 1 patient each occurred in the study group. In addition, one patient was hospitalized due to tachycardiac paroxysmal atrial fibrillation and transient ischemic attack. Twenty-nine (8.7% (29/336)) examinations in the study group were aborted because of either chest pain, dyspnea, nausea, dizziness, a drop of SBP, arrhythmias, tachycardiac paroxysmal atrial fibrillation, monomorphic PVCs, or non-sustained ventricular tachycardia. The rate of aborted examination was comparable to the control group (7.5% (15/(200), p = 0.631). Univariable logistic regression analysis revealed that female sex (odds ratio [OR] 2.21, 95% confidence intervals [CI] 1.2–4.3, p = 0.017) and inducible ischemia (OR 3.50, 95% CI 2.0–6.0, p < 0.001) were associated with an increased risk of side effects during DCMR.

**Conclusion:**

Dobutamine stress CMR did not show a relevant increase in adverse events in patients with prior CABG compared to patients without prior CABG. Female sex and dobutamine-induced myocardial ischemia are associated with side effects during DCMR.

## Graphical abstract

**Figure:**
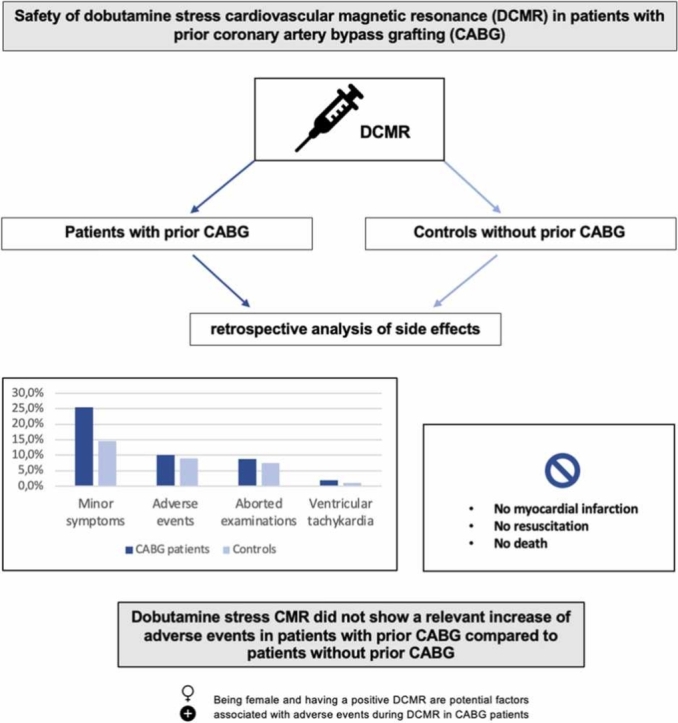
Dobutamine stress CMR did not show a relevant increase in adverse events in patients with prior CABG compared to patients without prior CABG. Female sex and dobutamine-induced myocardial ischemia are associated with side effects during DCMR

## 1 Background

Patients with severe coronary artery disease (CAD), despite successful coronary artery bypass grafting (CABG), are at increased risk of major adverse cardiac events [1]. Among imaging techniques, dobutamine stress cardiovascular magnetic resonance (DCMR) is highly sensitive and specific for detecting significant CAD [2], [3], [4], [5]. High diagnostic accuracy for the detection of angiographically defined CAD with a sensitivity of 0.83 (95% confidence intervals [CI]: 0.79–0.88) and a specificity of 0.86 (95% CI: 0.81–0.91) has been described previously for DCMR [6], [7]. DCMR is used to detect inducible wall motion abnormalities (WMA) and myocardial perfusion deficits as well as myocardial scar, which helps to identify patients at increased risk for cardiac death and myocardial infarction [8], [9], [10]. Additionally, it allows for cardiac risk stratification in patients with ischemic heart disease [10], [11]. Limited data exist on direct comparison of different stress agents in patients with prior CABG [12], [13]. Klein et al. showed a higher diagnostic accuracy of DCMR compared to adenosine stress perfusion in patients with prior CABG [13], although the question of higher diagnostic accuracy has still not been definitively clarified. The effect of vasoactive agents like adenosine on native coronary arteries is reproducible, while the vasoactive effect on grafts may vary [12], [14], [15]. Further, the increased length of graft conduits compared to native coronary arteries may complicate the interpretation of perfusion deficits [16], [17], [18]. Therefore, DCMR is preferred over adenosine in patients with prior CABG at our center. Dobutamine as a stress agent is generally considered safe [9], [10], [19], [20], [21]. However, severe adverse events such as myocardial infarction, sustained ventricular tachycardia (s-VT), and non-sustained ventricular tachycardia (ns-VT) have been reported during DCMR [19], [20]. Data on this modality’s safety in patients with prior CABG are scarce. Consequently, the objective of this study was to evaluate the safety of DCMR in patients who have undergone CABG.

## 2 Methods

### 2.1 Study design and study population

**Fig. 1:**
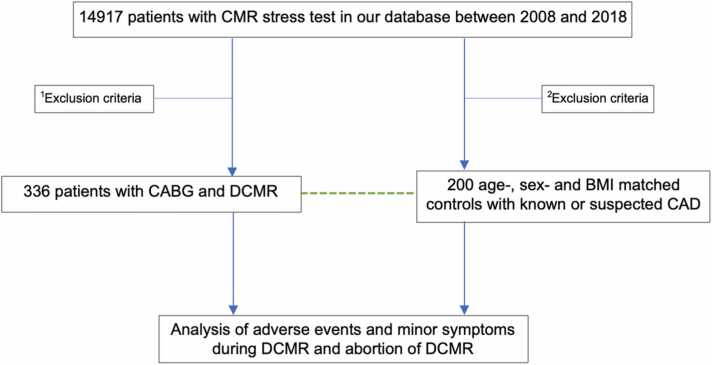
Selection of CABG patients and controls. ^1^Exclusion criteria: no history of CABG at the date of CMR stress test and adenosine stress test (only patients after DCMR were enrolled). ^2^Exclusion criteria: history of CABG at the date of CMR stress test and adenosine stress test (only patients after DCMR were enrolled). *BMI* body mass index, *CABG* coronary artery bypass grafts, *CAD* coronary artery disease, *CMR* cardiovascular magnetic resonance imaging, *DCMR* dobutamine stress cardiac magnetic resonance imaging

We conducted a retrospective study at the Department of Cardiology, Angiology, and Pneumology of the Heidelberg University Hospital on patients who had undergone DCMR after successful CABG between 2008 and 2018. Patients were identified from our local clinical database. Inclusion criteria were a history of CABG at the date of CMR stress test and usage of dobutamine as stressor. A control group of age-, sex-, and body mass index (BMI)-matched patients was enrolled, who had undergone DCMR, but had no prior CABG. We assessed cardiovascular risk factors (CVRF) such as arterial hypertension, hypercholesterolemia, diabetes mellitus, smoking history, and family history of cardiovascular disease using medical reports (Fig. 1).

### 2.2 CMR acquisition protocol

Patients preparation was done according to standardized CMR protocols [22]. CMR was performed in supine position as previously described [23], [24], [25], [26]. The study used 1.5T or 3T whole-body magnetic resonance imaging (MRI) scanners (Achieva, IngeniaCX, Ingenia, Philips Healthcare, Best, The Netherlands), equipped with a commercial cardiac phased array receiver coil. Cine long axis in two-, three- and four-chamber views and short-axis cine images covering the whole left ventricle (LV) from the annulus of the atrioventricular valves to the apex (8 mm slice thickness, no gap between each slice) were obtained. The imaging used a breath-hold, segmented-k-space balanced steady-state free precession sequence employing retrospective electrocardiogram or pulse oximetric gating with 40 (Achieva) or 35 (Ingenia/IngeniaCX) phases per cardiac cycles. For 1.5T, repetition time (TR) was 2.8 ms and echo time (TE) was 1.4 ms. Flip angle (FA) was 60°. For 3T, TR was 2.9 ms, TE was 1.4 ms, and FA was 45°. Breath-hold time was 7–10 seconds per acquisition using prospective gating. Data were analyzed using commercially available workstations (Viewforum and IntelliSpace Portal, ISP, Philips Healthcare, Best, The Netherlands) and the certified cmr42 software (Version 5.6.6, Circle Cardiovascular Imaging Inc., Calgary, Canada) for volumetric analysis. Ventricular volumes and left ventricular ejection fraction (LV-EF) were semi-automatically measured in short-axis stacks by manually tracing epi- and endocardial borders, excluding papillary muscles from the myocardium. Dobutamine stress CMR was performed as previously described [23], [27], [28], using cine long axes two-, three- and four-chamber views and three short-axis views (apical, mid-ventricular, and basal). Dobutamine was infused during 3-minute stages at incremental doses of 10, 20, 30, and 40 µg/kg of body weight/min until at least 85% of the age-predicted heart rate (220 age in years) was reached. Atropine was used in 0.25 mg increments (up to 2.0 mg) if the target heart rate was not reached. Perfusion imaging was performed during high-dose dobutamine stress CMR at maximum heart rate using a single-shot, turbo field gradient recalled echo-EPI (Echo-Planar-Imaging)sequence in three short-axis planes (apical, mid-ventricular, and basal). Images were acquired during the first pass of a 0.2 mmol/kg of body weight Magnevist (Bayer HealthCare, Berlin, Germany) or Gadovist (Bayer HealthCare, Leverkusen, Germany) 0.14 mmol/kg body weight (1.5T) or 0.1 mmol/kg body weight (3T) (after February 2016). Images were assessed for WMA and perfusion deficits. Stress testing was stopped when the target heart rate was reached. WMA were defined as a significant worsening of wall motion in at least one segment. Failure to reach 85% of age-predicted maximal heart rate was considered as a nondiagnostic result. Electrocardiographic rhythm, symptoms, and oxygen saturation were continuously monitored. Peripheral blood pressure was measured at rest, at every stage of dobutamine infusion, and after stress testing. Contraindications for dobutamine stress CMR were as previously described [22]: unstable angina pectoris, severe systemic arterial hypertension (≥220/120 mmHg), severe aortic valve stenosis (peak aortic valve gradient >60 mmHg or aortic valve area <1 cm^2^), complex cardiac arrhythmias including uncontrolled atrial fibrillation, hypertrophic obstructive cardiomyopathy, acute myocarditis, endocarditis or pericarditis, and uncontrolled congestive heart failure. Additionally, atropine was not used if the following contraindications were present: narrow-angle glaucoma, myasthenia gravis, obstructive uropathy, or gastrointestinal disorders. To ensure a high safety standard, all DCMR examinations in this study were performed by experienced magnetic resonance technologists and physicians, who closely monitored patients, with resuscitation equipment readily available.

### 2.3 Evaluation of side effects

Side effects were defined as adverse events and minor symptoms. Adverse events were defined as the following: death, myocardial infarction, unstable angina, s-VT, ns-VT, increase in systolic blood pressure (SBP) (>200 mmHg), decrease in SBP > 40 mmHg and other arrhythmias (premature ventricular contractions [PVC], left bundle-branch block, atrial fibrillation, supraventricular tachycardia, sinus tachycardia, bradycardia, triplets) as well as the need of hospitalization. Minor symptoms were defined as chest pain, nausea and emesis, headache, and dyspnea. Instances, in which DCMR had to be aborted, were also recorded. Furthermore, we compared patients with and without side effects.

### 2.4 Ethics approval

The retrospective analysis was approved by the local institutional ethics committee in accordance with the Declaration of Helsinki (S-151/2019). The requirement for individual informed consent was waived by the local institutional ethics committee.

### 2.5 Statistical analysis

For statistical analysis, we used IBM SPSS Statistics 24 (IBM Corp., Armonk, New York), with p < 0.05 taken to indicate statistical significance for all statistical tests. Group differences for continuous variables were tested using the independent t-test. Continuous and normally distributed variables are expressed as mean ± standard deviation. Continuous variables without normal distribution were tested using the Kolmogorov-Smirnov test. They are stated as median and interquartile range. Group differences for categorical variables were compared using chi-square test. Correlation analysis for the occurrence of adverse events was performed using Pearson correlation. Univariable logistic regression model was used to assess the association between each variable and the occurrence of adverse events. Results are reported as odds ratio (OR) with 95% CI and p < 0.05 taken to indicate statistical significance.

## 3 Results

### 3.1 Baseline characteristics

A total of 336 patients who underwent CABG (84.5% male (284/336), median age 69.8 ± 8.8 years) and 200 sex-, age-, and BMI-matched controls were included in the study. There were no significant differences regarding sex, age, BMI, and body surface area (BSA) between CABG patients and the control group. The prevalence of CVRF was high in both groups. Overall, CVRF were present in 99.7% (335/336) of CABG patients and 95.9% (192/200) of controls. CABG patients had a significantly higher occurrence of hypertension, hypercholesterolemia, and diabetes mellitus compared to controls (Table 1). The availability of information in the control group varied, with available information for hypertension in 59% of controls, for hypercholesterolemia in 59% (118/200) of controls, for diabetes mellitus in 55% (110/200) of controls, for smoking history in 53% (106/200) of controls, and for family history of CAD in 52% (104/200) of controls.

**Table 1:** Baseline characteristics and DCMR parameters.

	CABG (n = 336)	Controls (n = 200)	p
*Demographics*			
Male (n)	284 (84.5%)	167 (83.5%)	0.754
Age (years)	69.8 ± 8.8	70.7 ± 9.1	0.285
BMI (kg/m²)	27.6 ± 4	27.1 ± 3.7	0.173
BSA (m²)	1.97 ± 0.25	1.96 ± 0.2	0.852
*Cardiovascular risk factors*			
Hypertension (%)	94.9	82.2	<0.001
Hypercholesterolemia (%)	83.3	72.0	<0.01
Diabetes mellitus (%)	36.3	24.5	<0.05
History of smoking (%)	45.8	47.2	0.810
Family history of coronary artery disease (%)	37.5	27.9	0.073
Cardiovascular risk factors (%)*	99.7	95.9	<0.001
*DCMR parameters*			
Heart rate at maximum stress (bpm)	129.3 ± 15.4	132.1 ± 13.9	<0.05
Maximum dose of dobutamine (µg/kg/min)	38.5 ± 4.5	38.7 ± 3.8	0.712
Atropin used (%)	198 (58.9%)	119 (59.5%)	0.964
Atropin dose (mg)	0.4 ± 0.5	0.4 ± 0.5	0.800
Inducible ischemia (positive DCMR) (%)	85 (25.3%)	26 (13%)	<0.001
LV-EF (%)	52.9 ± 11.1	55.7 ± 11.6	<0.01

Baseline characteristics of patients after CABG and controls and DCMR parameters: results and use and dose of dobutamine and atropine. Values are mean ± SD or n (%)

*BMI* body mass index, *BSA* body surface area, *CABG* coronary artery bypass grafting, *DCMR* dobutamine stress cardiovascular magnetic resonance imaging, *LV-EF* left ventricular ejection fraction, *SD* standard deviation

* Patients who had at least one cardiovascular risk factor documented. Information for CVRF variables was available for 100% of CABG patients

### 3.2 Dobutamine stress cardiovascular magnetic resonance imaging

**Fig. 2:**
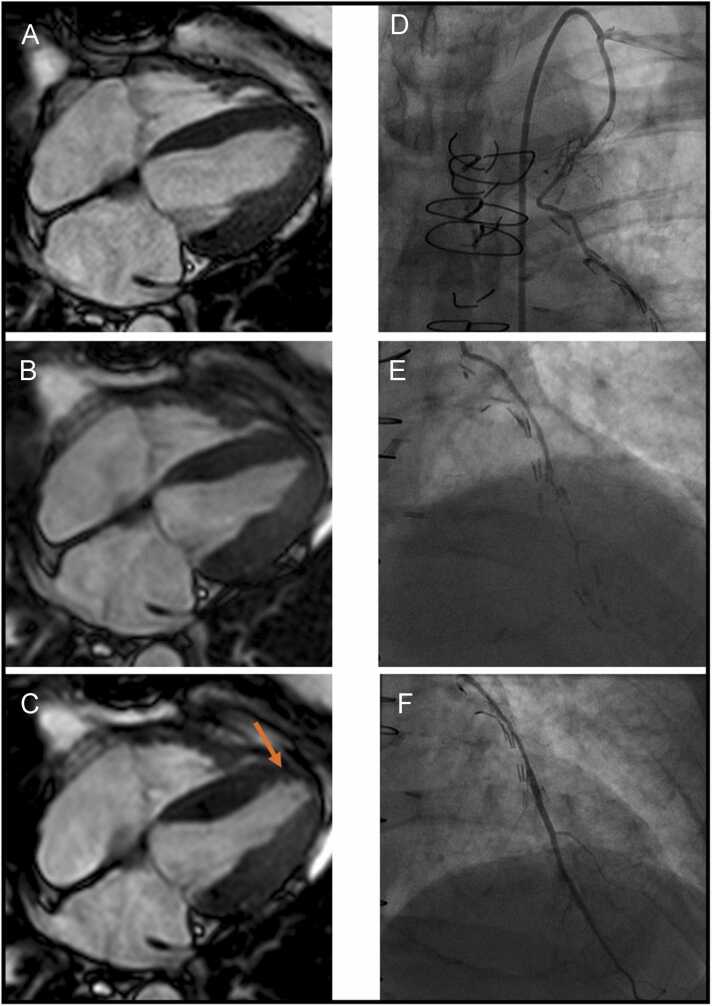
Representative DCMR-images and coronary angiography of a 66-year-old male patient after bypass surgery (LIMA to LAD, Y-bypass to LAD and LCX and venous bypass to RCA). Male patient after bypass surgery (LIMA to LAD, Y-bypass to LAD and LCX, and venous bypass to RCA) referred to DCMR. The patient was symptomatic with chest pain and dyspnea under exertion (Canadian Cardiovascular Society (CCS) grading of angina pectoris: Class III). Symptoms were reproducible under peak stress at DCMR. Left column: four-chamber views at end-systole at rest (A), at 30 µg/kg of body weight/min (B), and at 40 µg/kg of body weight/min (C). Orange arrow indicates inducible wall motion abnormalities (apical anterior, apical septal, and apex) at peak stress. Right column: coronary angiogram of the same patient. Proximal LIMA to LAD was open (D). Venous bypass to RCA was open. Y-bypass to LCX was open, subtotal stenosis of Y-bypass to LAD with a history of unsuccessful intervention. Stenosis of distal LIMA to LAD and LAD (E). Taking DCMR results with inducible WMA, suitable to the stenosis of LIMA to LAD and LAD into consideration, decision for treatment was made by our interventional team. Improved flow after implantation of two drug-eluting stents (at distal LIMA and at LAD) (F). *DCMR* dobutamine stress cardiovascular magnetic resonance, *LAD* left atrial descending coronary artery, *LCX* left circumflex coronary artery, *RCA* right coronary artery, DCMR dobutamine stress cardiovascular magnetic resonance, *LIMA* left internal mammary artery

During DCMR, there was no significant difference in the dose of dobutamine between the CABG and the control group, nor in the use of atropine(Table 1). CABG patients accomplished a slightly, but significantly less maximum heart rate at stress (Table 1). Further, significantly more CABG patients (20.2% (68/336)) failed to meet the diagnostic threshold of 85% predicted max heart rate compared to controls (9.% (18/200), p < 0.05). CABG patients showed a significantly higher incidence of inducible ischemia than controls. Additionally, the LV-EF was significantly lower in CABG patients compared to controls (Table 1). Fig. 2 shows the exemplary result of a positive stress exam of a 66-year-old male patient after CABG and with inducible WMA (apical anterior, apical septal, and apex). It further shows the coronary angiography with a stenosis of distal LIMA (Left Internal Mammary Artery(Backspace) to left atrial descending coronary artery (LAD) and LAD of the same patient before and after stenting.

### 3.3 Adverse events

Adverse events occurred in 35 CABG patients (10% (35/336)) and in 18 controls (9% (18/200)) without a significant difference between the groups (p = 0.595) (Table 2). No myocardial infarction, resuscitation, or death due to DCMR occurred in either of the groups. One patient from each group required hospitalization after DCMR. Hospitalization occurred due to tachycardic atrial fibrillation with a transient ischemic attack in a CABG patient, while one of the controls required hospitalization due to s-VT. S-VT occurred in two patients (1% (2/200)) of the control group, while none of the CABG patients suffered from s-VT (p = 0.066). Ns-VT occurred in six CABG patients, whereas none of the controls exhibited ns-VT (p = 0.057). A significant increase in SBP occurred in five CABG patients (1.5% (5/336)) and in two patients (1% (2/200)) in the control group (p = 0.625). A significant decrease in SBP was observed in 12 CABG patients (3.6% (12/336)) compared to 7 patients (3.5% (7/200)) in the control group (p = 0.947). Other arrhythmias occurred in 11 CABG patients (3.3% (11/336)) and in 6 controls (3% (6/200)) (p = 0.861). The various other arrhythmias in the CABG group included monomorphic PVC in two patients, bigeminy in two patients, left bundle-branch block in two patients, as well as tachycardiac paroxysmal atrial fibrillation, bradycardia, supraventricular tachycardia, couplets, triplets, and sinus arrhythmia in one patient each. No new atrioventricular (AV)-block was observed during DCMR.

**Table 2:** Adverse events and minor symptoms during DCMR and abortion of DCMR.

	CABG	Controls	p
*Adverse events*			
Overall	35 (10%)	18 (9%)	0.595
Sustained VT	0	2 (1%)	0.066
Non-sustained VT	6 (1.8%)	0	0.057
Hospitalization	1 (0.3%)	1 (0.5%)	0.713
Decrease in SBP >40 mmHg	12 (3.6%)	7 (3.5%)	0.947
Increase in SBP >200 mmHg	5 (1.5%)	2 (1%)	0.625
Other arrhythmias	11 (3.3%)	6 (3%)	0.861
**Other arrhythmias included:**			
Monomorphic premature ventricular contractions	2 (0.6%)	0	0.274
Bigeminy	2 (0.6%)	1 (0.5%)	0.886
Left bundle-branch block	2 (0.6%)	0	0.274
Atrial fibrillation	1 (0.3%)	2 (1%)	0.292
Supraventricular tachycardia	1 (0.3%)	3 (1.5%)	0.118
Sinus arrhythmia	1 (0.3%)	0	0.440
Bradycardia	1 (0.3%)	0	0.440
Triplets	1 (0.3%)	0	0.440
*Minor symptoms*			
Overall	86 (25.6%)	29 (14.5%)	<0.05
Chest pain	73 (21.9%)	18 (9.0%)	<0.001
Dyspnea	18 (5.4%)	6 (3.0%)	0.197
Nausea (and emesis)	7 (2.1%)	5 (2.5%)	0.760
Dizziness	2 (0.6%)	1 (0.5%)	0.882
Headache	2 (0.6%)	1 (0.5%)	0.882
Claustrophobia	1 (0.3%)	0	0.440
*Abort*ed *DCMR*			
Overall	29 (8.7%)	15 (7.5%)	0.630
**Reasons for abortion of DCMR:**			
Minor symptoms (abortion on patients request)	25 (86.2%)	11 (73.3%)	0.294
Non-sustained VT	1 (3.4%)	0	0.467
Sustained VT	0	1 (6.7%)	0.160
Other arrhythmia	2 (6.8%)	1 (6.7%)	0.977
Decrease in SBP	1 (3.4%)	0	0.467
Increase in SBP	0	2 (13.3%)	<0.05

Adverse events and minor symptoms during DCMR and abortion of DCMR. Values are n (%)

*CABG* coronary artery bypass grafting, *DCMR* dobutamine stress cardiovascular magnetic resonance imaging, *SBP* systolic blood pressure, *VT* ventricular tachycardia

### 3.4 Minor symptoms

Minor symptoms were reported in 86 CABG patients (25.6% (86/336)) and 29 controls (14.5% (29/200)) (p < 0.05). Among these symptoms, chest pain was the most frequently reported and occurred significantly more often in CABG patients compared to controls. Other symptoms observed in CABG patients during DCMR included dyspnea, nausea, dizziness, headache, and claustrophobic attack. Except for chest pain, there were no significant differences between the groups in terms of minor symptoms (Table 2).

### 3.5 Aborted DCMR

DCMR had to be aborted in 29 CABG patients (8.7% (29/336)) and 15 controls (7.5% (15/200)) (p = 0.630). The most common reason for termination was the presence of non-tolerable minor symptoms with abortion upon patients' request (25 patients), in particular chest pain (20 patients). Other reasons were s-VT, ns-VT, other arrhythmias, and a decrease in SBP. Notably, in controls, an increase in SBP was significantly more often the reason for termination compared to CABG patients. However, this occurred in only two controls. Except for an increase in SBP, there were no significant differences between the groups in terms of reasons for the abortion of DCMR (Table 2).

### 3.6 Comparison of CABG patients with and without side effects

Among CABG patients, those who experienced side effects during DCMR were significantly more often female. The rate of inducible ischemia (positive stress CMR) was significantly higher in CABG patients with side effects compared to CABG patients without side effects (Table 3). However, there were no significant differences in terms of age or BMI between the two groups. There were no significant differences regarding CVRF or comorbidities (number of CABG, severity of CAD, prior ST-elevation myocardial infarction [STEMI] or non-ST elevation myocardial infarction [NSTEMI], and prior stroke or transient ischemic attack). Furthermore, no significant differences were observed in CMR parameters such as LV-EF, LV end-systolic volume (ESV) or LV end-diastolic volume (EDV), heart rate, or systolic or diastolic blood pressure. Overall, 12.6% (42/333) of the patients had only arterial bypass grafts, 14.1% (47/333) had only venous bypass grafts, and 73.3% (244/333) had both. In the group of patients with side effects, 8.3% (9/109) had only arterial bypass grafts, 12.8% (14/109) had only venous bypass grafts, and 78.9% (86/109) had both. In the group of patients without side effects, 14.7% (33/225) had only arterial bypass grafts, 14.7% (33/225) had only venous bypass grafts, and 70.7 (159/225) had both. There was no correlation between the type of bypass graft and the occurrence of side effects (p = 0.197).

**Table 3:** Comparison of CABG patients with and without side effects during dobutamine stress cardiac magnetic resonance imaging.

	Patients with side effects (n = 109)	Patients without side effects (n = 227)	p
*Demographics*			
Age (years)	69.1 ± 9	70.1 ± 9	0.338
Female sex (%)	22.0	12.4	<0.05
BMI (kg/m²)	27.5 ± 4	27.6 ± 4	0.690
*Cardiovascular risk factors*			
Hypertension (%)	97.2	94.2	0.225
Hypercholesterolemia (%)	85.3	82.2	0.477
Diabetes mellitus (%)	36.0	36.7	0.901
History of smoking (%)	44.0	46.2	0.707
Family history of CAD (%)	35.6	41.3	0.310
*Comorbidities*			
Number of CABG (n)	3 (2–4)	3 (3–4)	0.074
Severity of CAD* (n)	3 (3)	3 (3)	0.243
Prior STEMI (%)	29.8	27.5	0.670
Prior NSTEMI (%)	24.9	24.0	0.976
Prior stroke/transitory ischaemic attack (%)	9.2	9.3	0.963
*DCMR parameters*			
LV-EF (%)	54.4 ± 11	52.1 ± 11	0.073
LV-ESV (%)	80.4 ± 38	87.8 ± 47	0.152
LV-EDV (%)	170.6 ± 45	175.8 ± 54	0.385
Heart rate at rest (bpm)	64.3 ± 11	65.8 ± 10	0.228
BP systolic at rest (mmHg)	128.7 ± 21	126.4 ± 18	0.288
BP diastolic at rest (mmHg)	67.5 ± 14	66.1 ± 11	0.329
Inducible ischemia (positive stress CMR) (%)	41.3	17.8	<0.001

Comparison of patients with CABG with and without side effects during DCMR. Values are mean ± SD, median (interquartile range) or n (%)

*BMI* body mass index, *BP* blood pressure, *CABG* coronary artery bypass grafting, *CAD* coronary artery disease, *CMR* cardiovascular magnetic resonance, *DCMR* dobutamine stress cardiovascular magnetic resonance, *EF* ejection fraction, *ESV* end-systolic volume, *EDV* end-diastolic volume, *LV* left ventricular, *SD* standard deviation, *STEMI* ST-elevation myocardial infarction, *NSTEMI* non-ST elevation myocardial infarction

* Severity of CAD = one-, two-, and three-vessel disease

### 3.7 Assessment of univariable model for predicting the occurrence of side effects during DCMR

Univariable logistic regression analysis revealed that female sex and inducible ischemia were associated with the occurrence of side effects (Table 4).

**Table 4:** Univariable analysis for the prediction of side effects during DCMR.

	OR	95% CI	p
*Patients characteristics*			
Age		−0.641 to 2.058	0.302
Female sex	2.208	1.154 to 4.225	<0.05
BMI		−0.017 to 0.011	0.306
*Cardiovascular risk factors*			
Hypertension		0.586–9.425	0.228
Hypercholesterolemia		0.640–2.461	0.508
Diabetes mellitus		0.614–1.713	0.923
History of smoking		0.675–1.836	0.674
Family history of CAD		0.700–1.936	0.558
*Comorbidities*			
Prior STEMI		0.629–1.857	0.777
Prior NSTEMI		0.458–1.451	0.487
Prior stroke /TIA		0.431–2.322	0.998
Atrial fibrillation		0.323–1.123	0.110
Number of CABG		−0.014–0.091	0.151
Severity of CAD*		−0.126–0.179	0.733
*DCMR parameters*			
LV-EF		−0.012 to 0.016	0.740
LV-EDV		−0.004 to 0.004	0.854
Heart rate		−0.009 to 0.002	0.267
BP systolic		−0.002 to 0.004	0.558
BP diastolic		−0.003 to 0.007	0.482
Inducible ischemia	3.502	2.044–6.001	<0.001

Univariable logistic analysis model for the prediction of the occurrence of side effects during DCMR in patients with prior CABG

*BMI* body mass index, *BP* blood pressure, *CABG* coronary artery bypass grafting, *CAD* coronary artery disease, *CI* confidence interval, *CMR* cardiovascular magnetic resonance, *DCMR* dobutamine stress cardiovascular magnetic resonance, *EF* ejection fraction, *EDV* end-diastolic volume, *LV* left ventricular, *NSTEMI* non-ST elevation myocardial infarction, *OR* odds ratio, *STEMI* ST-elevation myocardial infarction, *TIA* transient ischemic attack

* Severity of CAD = one-, two-, and three-vessel disease

### 3.8 Comparison of female and male patients

Two hundred and eighty-four male and 52 female patients with CABG who underwent DCMR were enrolled. There were no significant differences in age or BMI between male and female patients. In terms of CVRF, there were no significant differences in the prevalence of hypertension, hypercholesterolemia, or diabetes mellitus. Notably, male patients had a significantly higher prevalence of a smoking history compared to female patients. Conversely, female patients had a significantly higher prevalence of a family history of CAD. Regarding cardiovascular comorbidities, there were no significant differences between male and female patients in severity of CAD, prior STEMI, prior NSTEMI, or prior stroke or TIA. However, the number of CABG was significantly higher in male patients. Further, female patients had a significantly higher LV-EF. There was no significant difference between male and female patients regarding inducible ischemia (Table 5). Female patients had a significantly higher prevalence of a drop in SBP compared to male patients. The hospitalized CABG patient (due to tachycardic atrial fibrillation with transient ischemic attack) was also female (Table 5). There was no difference between male and female patients in ns-VT, increase in SBP, or other arrhythmias. Further, regarding minor symptoms, the prevalence of dyspnea and nausea and emesis was significantly higher in female CABG patients (Table 5). There were no significant differences in the prevalence of chest pain, dizziness, headache, or claustrophobia (Table 5).

**Table 5:** Comparison of female and male patients with prior CABG.

	Male patients with prior CABG (n = 284)	Female patients with prior CABG (n = 52)	p
*Demographics*			
Age (years)	69.6 ± 8.7	71.0 ± 9.1	0.291
BMI (kg/m²)	27.7 ± 3.9	26.8 ± 4,2	0.150
*Cardiovascular risk factors*			
Hypertension (%)	95.4	92.3	0.346
Hypercholesterolemia (%)	84.2	78.8	0.345
Diabetes mellitus (%)	36.6	34.6	0.782
History of smoking (%)	48.2	32.7	0.039
Family history of CAD (%)	34.9	51.9	0.019
*Comorbidities*			
Number of CABG (n)	3 (2–4)	3 (2–3)	0.030
Severity of CAD* (n)	3 (3)	3 (3)	0.898
Prior STEMI (%)	29.6	25.0	0.503
Prior NSTEMI (%)	43.5	26.9	0.606
Prior stroke/transitory ischaemic attack (%)	9.2	9.6	0.916
*DCMR parameters*			
LV-EF (%)	51.9 ± 11.1	58.0 ± 9.3	<0.001
Heart rate at rest (bpm)	65.1 ± 10.2	66.4 ± 11.5	0.385
BP systolic at rest (mmHg)	126.8 ± 17.3	129.0 ± 25.6	0.444
BP diastolic at rest (mmHg)	67.5 ± 11.4	61.3 ± 14.7	<0.01
Inducible Ischemia (positive stress CMR) (%)	26.2	21.2	0.447
*Adverse events*			
Ns-VT	6 (2.1%)	0 (0%)	0.288
Hospitalization	0 (0%)	1 (1.9%)	0.020
Drop in SBP >40 mmHg	6 (2.1%)	6 (11.1%)	0.001
Increase in SBP >200 mmHg	5 (1.8%)	0 (0%)	0.333
Other arrhythmias	6 (2.1%)	2 (3.7%)	0.456
*Minor symptoms*			
Chest pain	64 (22.5%)	9 (16.7%)	0.388
Dyspnea	12 (4.2%)	6 (11.1%)	<0.05
Nausea (and emesis)	2 (0.7%)	5 (9.3%)	<0.001
Dizziness	1 (0.4%)	1 (1.9%)	0.178
Headache	1 (0.4%)	1 (1.9%)	0.178
Claustrophobia	1 (0.4%)	0 (0%)	0.667

Comparison of female and male patients. Values are mean ± SD, median (interquartile range), or n (%)

*BMI* body mass index, *BP* blood pressure, *CABG* coronary artery bypass grafting, *CAD* coronary artery disease, *CMR* cardiovascular magnetic resonance, *DCMR* dobutamine stress cardiovascular magnetic resonance, *EF* ejection fraction, *LV* left ventricular, *NSTEMI* non-ST elevation myocardial infarction, *ns-VT* non-sustained ventricular tachycardia, *SBP* systolic blood pressure, *STEMI* ST-elevation myocardial infarction

* Severity of CAD = one-, two-, three-vessel disease

## 4 Discussion

In this single-center study, we aimed to assess the safety of dobutamine stress CMR in patients with severe CAD who had undergone CABG. Our study included patients with a high burden of significant CAD, aiming to evaluate the safety of dobutamine stress CMR in this population. Interestingly, the high-risk group of patients with CABG tolerated dobutamine stress CMR well. Incidental arrhythmias occurred at a similar rate compared to the control group. We recorded hospitalizations in one CABG patient due to tachycardic atrial fibrillation with transient ischemic attack. These results indicate a comparable safety profile in patients with prior CABG when compared to a matched control group and previous studies, where dobutamine as a stress agent was generally considered safe [9], [10], [19], [20], [21].

We observed a significantly higher occurrence of minor symptoms in CABG patients undergoing DCMR compared to control group, resulting in a slightly higher rate of DCMR abortions within the CABG group. The higher incidence of chest pain and other minor symptoms during DCMR in CABG patients can be attributed to the higher burden of significant CAD. In our study, CABG patients had a known severe CAD, with the majority having a three-vessel CAD (91% (306/336)) and having already undergone three (40% (134/336)) or four (23% (77/336)) distal anastomoses. On the other hand, the controls were investigated for known or suspected relevant CAD and likely had a less severe CAD than the CABG patients. The higher incidence of minor symptoms during DCMR in CABG patients aligns with the higher incidence of positive stress tests in this group compared to controls (CABG: 25.3% (85/336), controls: 13.0% (26/200), p < 0.001). Additionally, the observed incidence of chest pain in a dobutamine stress echocardiography study on 1118 patients was 19.3% and is therefore comparable to the observed incidence in our study [29].

The rate of aborted DCMR observed in our study is consistent with the rates reported in previous studies, which ranged from 3.0% to 11.0% [10], [19], [21]. While there were more minor symptoms in CABG patients compared to controls, it is important to note that no myocardial infarction, resuscitation, or death occurred in either group. Ventricular tachycardia occurred in 1.8% (6/336) of CABG patients and 1% (2/200) of the controls without a statistically significant difference. Previously, Wahl et al. reported an incidence of ventricular tachycardia of 0.5% in 1075 dobutamine stress CMR examinations, along with transient second-degree AV-blocks with a 2:1 conduction ratio in 0.2% of the examinations, which was not observed in our study [19]. Other previous studies have reported rates of major adverse events such as life-threatening arrhythmias, myocardial infarctions, or death up to 1% [10], [19], [21], [30], [31]. However, indication for DCMR should be considered carefully and as recommended, patients should always be closely monitored and resuscitation equipment, including external defibrillator, must be available [22].

### 4.1 Identification of CABG patients at risk for side effects

The comparison between CABG patients with and without side effects during dobutamine stress CMR revealed that female patients had a significantly higher risk of experiencing side effects. There were no further significant differences regarding baseline characteristics, CVRF, comorbidities, or cardiac function between CABG patients with and without side effects. Univariable regression analysis showed that female patients had an OR of 2.21 for side effects. Especially the prevalence of a drop in SBP, dyspnea and nausea and emesis, was significantly higher in female patients compared to male patients. Further, the only hospitalized CABG patient was female. The comparison of male and female patients did not show significant differences regarding baseline characteristics. Both groups had a similar severity of CAD with a slightly but significantly higher number of CABG in male patients. LV-EF was in the lower sex-specific normal range in both groups [32]. Also, there was no significant difference in the prevalence of inducible ischemia (positive stress CMR) between male and female patients. A more severe CAD as a reason for the higher prevalence of adverse events in female patients seems unlikely. Previous studies using dobutamine stress echocardiography revealed that the incidence of stress-induced side effects such as nausea, headache, and anxiety was higher in women [33]. Other previous DCMR studies did not show a correlation between side effects due to dobutamine stress test and female sex [31]. However, previous studies did not explicitly investigate CABG patients.

Additionally, patients with side effects were found to have a significantly higher incidence of inducible ischemia (positive stress CMR) compared to patients without side effects. Univariable regression analysis showed that CABG patients with inducible ischemia had an OR of 3.50 for side effects. This suggests that side effects, particularly chest pain, are induced primarily by myocardial ischemia in patients with a positive dobutamine stress CMR. These findings are consistent with previous studies by Monmeneu Menadas et al., who showed an association between inducible ischemia and complications in 554 dobutamine stress CMR cases [31]. Overall, predicting the risk of side effects during DCMR in high-risk patients is challenging.

## 5 Limitations

The study has several limitations that should be taken into consideration. First, the study was conducted at a single center, which may limit the generalizability of the findings to other populations. Also, medical records and observations of side effects were retrospectively reviewed, which introduces the possibility of incomplete or inaccurate data collection. According to standardized CMR protocol, patients were instructed to pause ß-blockers and nitrates 12–24 hours before the examination due to potential of interaction with the stress agents [22]. Due to the retrospective data, there is no valid information about how many patients did not stop medication contrary to our recommendation. Further, more CABG patients failed to meet the diagnostic threshold of 85% predicted max heart rate compared to controls, which may represent an inadequate dobutamine challenge that affects the occurrence of side effects. In future studies, this should also be considered as a potential implication for the tests sensitivity to detect obstructive CAD in CABG patients. Prospective studies and standardized assessment of side effects would provide more robust and reliable results. Furthermore, the severity of CAD and CVRF was not known for all control patients, which limits the ability to accurately compare the CABG group with the control group. Also, patients in this study mostly had a normal or mildly reduced EF. It is unknown whether the results are transferable to patients with severely reduced EF, although reduced EF was not associated with a higher incidence of adverse events while DCMR in this study. Previous data suggest that perfusion stress CMR using adenosine or regadenoson was associated with significantly less complications and side effects compared to dobutamine stress CMR [23], [31]. As we have focused on dobutamine stress CMR in patients with severe CAD, we have not included patients who underwent perfusion stress CMR as a study or control group. Limited data exist on comparison of different stress agents in patients with prior CABG. Klein et al. showed a higher diagnostic accuracy for DCMR compared to adenosine stress perfusion in patients after CABG [13], although the question of higher diagnostic accuracy has still not been definitively clarified. However, evaluation of the prognostic value of different stress agents in patients with prior CABG may be needed to provide accurate recommendations whether the potentially increased rate of adverse events in DCMR compared to perfusion stress CMR is acceptable due to the higher diagnostic accuracy. At our center, based on our experience, we use DCMR in patients with prior CABG. Given the limitation of the sample size and potential selection bias, larger cohorts or multicenter studies may be necessary to provide more definite conclusions.

## 6 Conclusion

Dobutamine stress CMR did not show a relevant increase in adverse events in patients with prior CABG compared to patients without prior CABG. The absence of myocardial infarction or fatal adverse events during DCMR indicates a low risk of severe adverse events even in CABG patients. The study highlights that being female and having inducible myocardial ischemia are potential factors associated with side effects during DCMR in CABG patients. However, indication for DCMR should be considered carefully and DCMR should be performed under close patient monitoring.

## Funding

Not applicable.

## Author contributions

Conceptualization: J.H., J.S., A.O., and H.S.; study design: J.H. and J.S.; data acquisition: J.H., J.S., A.K., and H.H.; data interpretation: J.H.; first manuscript draft: J.H., J.S., D.L., and H.S.; manuscript revision: J.H., J.S., A.K., A.O., L.D.W., H.H., N.F., E.G., D.L., F.A., and H.S. All authors read and approved the final manuscript.

## Ethics approval and consent

The ethical committee of the University of Heidelberg approved this study (S-151/2019). Additional informed consent was waived. The study followed the Declaration of Helsinki (1964) and its later amendments.

## Consent for publication

Not applicable.

## Declaration of competing interests

The authors declare that they have no known competing financial interests or personal relationships that could have appeared to influence the work reported in this paper.

## Data Availability

The datasets used and analyzed during the current study are available from the corresponding author upon reasonable request.
